# Human Viral Oncoproteins and Ubiquitin–Proteasome System

**DOI:** 10.1055/s-0044-1790210

**Published:** 2024-09-02

**Authors:** Zahra Rafiei Atani, Sareh Sadat Hosseini, Hossein Goudarzi, Ebrahim Faghihloo

**Affiliations:** 1 Department of Microbiology, Faculty of Medicine, Shahed University, Tehran, Iran; 2 Student Research Committee, Faculty of Medicine, Shahed University, Tehran, Iran; 3 Reference Health Laboratory, Ministry of Health and Medical Education, Tehran, Iran; 4 Department of Microbiology, School of Medicine, Shahid Beheshti University of Medical Sciences, Tehran, Iran

**Keywords:** proteasome, UPS, ubiquitin, oncoprotein, tumor virus

## Abstract

Some human cancers worldwide may be related to human tumor viruses. Knowing, controlling, and managing the viruses that cause cancers remain a problem. Also, tumor viruses use ubiquitin–proteasome system (UPS) that can alter host cellular processes through UPS. Human tumor viruses cause persistent infections, due to their ability to infect their host cells without killing them. Tumor viruses such as Epstein–Barr virus, hepatitis C virus, hepatitis B virus, human papillomaviruses, human T cell leukemia virus, Kaposi's sarcoma-associated herpesvirus, and Merkel cell polyomavirus are associated with human malignancies. They interfere with the regulation of cell cycle and control of apoptosis, which are important for cellular functions. These viral oncoproteins bind directly or indirectly to the components of UPS, modifying cellular pathways and suppressor proteins like p53 and pRb. They can also cause progression of malignancy. In this review, we focused on how viral oncoproteins bind to the components of the UPS and how these interactions induce the degradation of cellular proteins for their survival.

## Introduction

An estimated 12 to 20% of all human cancers worldwide may be related to human tumor viruses. Despite their high prevalence, knowing, controlling, and managing the viruses that cause cancers remain a major problem. 1 Early studies showed that tumor viruses use ubiquitin–proteasome system (UPS) for many years. These viruses alter host cellular processes through UPS, e.g., life cycle, viral gene expression, and cause infection. 2 Studies showed that viruses can cause cancer due to their strong oncogenes, but human oncoviruses cannot cause cancer by themselves. They only cause viral infections and require cofactors to cause cancer, such as suppressing the host immune system, surrounding mutants, and chronic inflammations in different parts of the body. For proliferation, the virus uses host cell systems and proteins. Unlike other viruses, human tumor viruses cause persistent infections, as they can infect their host cells without killing them.

The viruses that cause human cancer are hepatitis B virus (liver cancer), papillomaviruses (cervical and other anogenital cancers), Epstein–Barr virus (EBV; Burkitt's lymphoma and nasopharyngeal carcinoma [NPC]), Kaposi's sarcoma-associated herpesvirus (KSHV, Kaposi's sarcoma [KS]), and human T cell lymphotropic virus (adult T cell leukemia [ATL]). They lead the cell to cancer through their oncoproteins that interact with the UPS and degrade tumor suppressor proteins like p53, pRb, or other proteins. In this review, we focused on how tumor viruses utilize the UPS to promote the degradation of cellular proteins for their survival.

## The Ubiquitin–Proteasome System

Proteasome is a large protein complex, which degrades intracellular proteins. The 26S proteasome contains two particles: a 20S core particle (CP) and one or two 19S regulatory particles (RP). This proteasome causes ubiquitin-dependent and energy-dependent degradation of specific substrates. 3

The ubiquitin–proteasome proteolytic pathway involves two steps: (1) attachment of the substrate by covalent connections of multiple ubiquitin molecules; (2) degradation of the attached protein by the 26S proteasome complex with the release of ubiquitin. This process is mediated by ubiquitin-recycling enzymes. 4

The ubiquitin-conjugating machinery is composed of E1, E2, E3, and E4 proteins. E1 activates ubiquitin, to generate a high energy. Then, the ubiquitin activated by E1 is transferred to E2. E2s catalyze covalent connections of ubiquitin to target proteins or transfer the activated ubiquitin to a high-energy E3 ubiquitin intermediate. E3s, which are responsible for the specific recognition of the many substrates of the ubiquitin system, display the greatest variety among its different components. E3s are a member of the ubiquitin–protein ligase family, and they are composed of two major domains: HECT domain and RING finger. 5 E4 is a ubiquitin chain elongation factor. This protein is identical to Ufd2 (ubiquitin fusion degradation pathway). 6 E4 specifies the protein family that shares a modified form of the RING finger, designated as U box. 4

Ubiquitin-like proteins (UBLs) show very little resemblance to ubiquitin. UBLs are divided into two groups: (1) those that can form an isopeptide bond posttranslationally with an internal lysine of a substrate. This group includes the SUMO/Sentrin/Smt3p/ISG15/FAT10 family of proteins and NEDD8/ Rub1 proteins. (2) The second class is larger proteins that contain a UBL domain. This group includes Rad23/HHR23A/HHR23B, Dsk2/hPLIC1/hPLIC2, and Bag-1 can bind to the proteasome. 7

Modification of proteins by covalent connections of ubiquitin or ubiquitin-related polypeptides and the degradation of some of the conjugates by the proteasome assist in the regulation of primary cellular processes degradation of a protein via the UPS. 4

## Epstein–Barr Virus

EBV is a largely nonpathogenic virus most important in all human populations, which infects more than 90% of the world's population. EBV is a gamma-herpesvirus, which is closely associated with several lymphomas (endemic Burkitt's lymphoma, Hodgkin's lymphoma, and nasal NK/T-cell lymphoma) and epithelial cancers (NPC and gastric carcinoma). 8

EBV encodes several proteins to avoid immune detection and elimination through inhibition of the UPS. In immunological evasion, EBNA-1 is the only EBV protein expressed. The glycine–alanine repeat (GAr) domain of EBNA-1 in latently infected B cells was shown to be the key element in preventing MHC class I presentation to CTL. 9

**Fig. 1:**
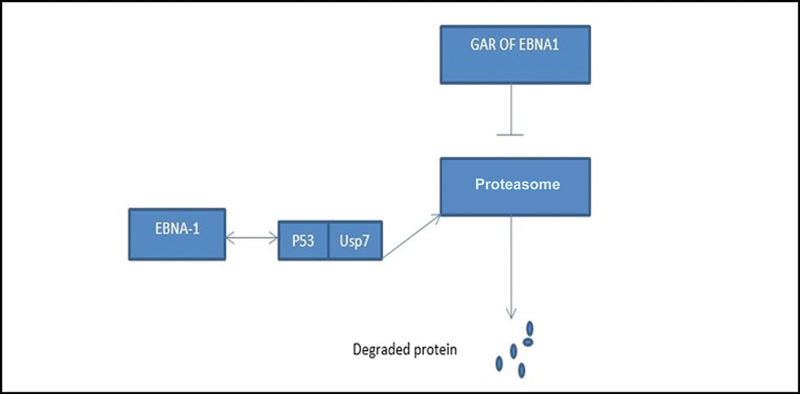
EBNA-1 associates with USP7 via binding to p53. p53 is blocked by USP7 and then degraded via proteasome.

Dantuma et al suggested that inhibition of proteasomal degradation by the Gly–Ala repeat of EBV virus is influenced by the length of the repeat and the strength of the degradation signal. 10 On the other hand, EBNA-1 associates with ubiquitin-specific protease 7 (USP7), by binding to the same pocket that p53 binds. Afterward, the deubiquitination of p53 by USP7 is blocked, leading to its degradation and, consequently, apoptosis inhibition ( Fig. 1 ). 11 BDLF3, a late lytic protein induces ubiquitination and downregulation of major histocompatibility complex class I (MHC-I) and MHC-II. 12

In addition, three proteins of EBV such as BNRF2, EBNA1, and BZLF1 could also disrupt the PML (promyelocytic leukemia) nuclear bodies then they evade the intrinsic antiviral response through the proteasome-dependent and independent mechanisms. 13 In the modulation of cell cycle checkpoints, EBNA-3 proteins, especially 3A and 3C, are investigated to play a role in the manipulation of the cell cycle. 14

Epstein–Barr nuclear antigen-3C (EBNA-3C) is important for the transformation, proliferation, and survival of infected B cells. This protein represses the transcription of p16INK4A, which is a cyclin-dependent kinase (CDK) inhibitor, therefore stabilizing the cyclin D1/CDK6 complex, and allowing proteasomal degradation of pRb and consequently cell cycle deregulation. 15

**Fig. 2:**
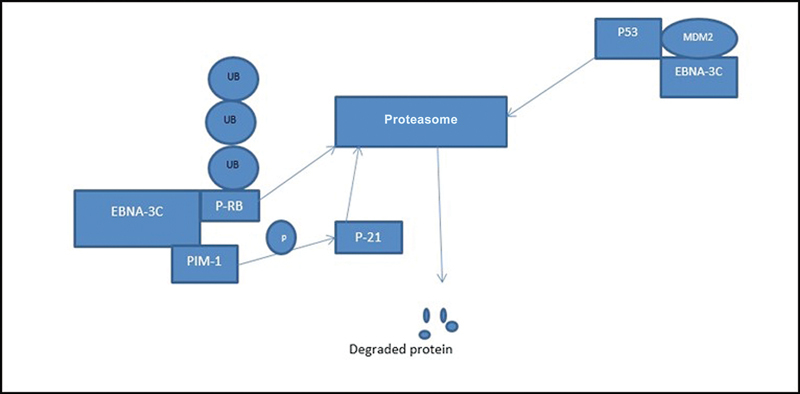
EBNA-3C associates with Pim-1 and then enhances the proteasomal degradation.

EBNA-3C physically interacts with proto-oncogene serine/threonine–protein kinase (Pim-1), which in turn phosphorylates p21 and enhances polyubiquitination of p21 and cell cycle deregulation (Bypass G1 arrest) and also apoptosis inhibition ( Fig. 2 ). 16 In addition, EBNA-3C enhances the phosphorylation and proteasomal degradation of p27KIP1 through SCFSkp2 E3-ubiquitin ligase and lead to cell cycle deregulation (bypass G1 and G2/M arrest). 17

Touitou et al showed that EBV virus EBNA3 proteins bind to the C8/a7 subunit of the 20S proteasome and are degraded by 20S proteasomes in vitro but are very stable in latently infected B cells. 18 Zancai et al have demonstrated that retinoic acid stabilizes p27Kip1 in EBV-immortalized lymphoblastoid B cell lines through increased proteasome-dependent degradation of the p45Skp2 and Cks1 proteins. 19

Moreover, EBNA-3C specifically interacts with Bcl-6 and promotes its ubiquitination and proteasomal degradation and subsequently cell cycle deregulation (bypass G1 arrest) and apoptosis inhibition (release of Bcl-2). 20 EBNA-3C stabilizes MDM2 E3 ligase for proteasomal degradation of p53 and leads to apoptosis inhibition. 21 In addition to EBNA-3, LMP-1 is one of the EBV latent proteins also. This protein possesses strong oncogenic activity through the activation of NFκB pathway. LMP-1 Induces proteolysis of p100 to p52 through proteasome and activates the noncanonical NFκB pathway and, consequently, apoptosis inhibition. 22

The first virally encoded proteins expressed after B cell infection are EBNA-5 and EBNA-2. EBNA-5 can combine the EBNA-2 during the activation of LMP-1 and Cp promoter of EBV virus. 23

In inhibition of apoptosis, EBV activates the noncanonical NFκB pathway, which in turn promotes the expression of inhibitor of apoptosis proteins (IAP), including XIAP, cIAP-1, and c-IAP-2 for cell survival through the action of LMP-1. 22 Also, EBNA-3C induce proteasomal degradation of Bcl-6, releasing the antiapoptotic protein, Bcl-2 from suppression by Bcl-6. 20

Therefore, the replication of EBV in EBV-infected cancer cells requires the manipulation of the host's UPS via the action of numerous viral proteins to intercede evasion of immune surveillance, interruption of cell cycle regulation, and inhibition of apoptosis. As a result, using proteasome inhibitors against EBV virus-associated cancers are therapeutic strategies.

## Hepatitis C

Hepatitis C virus (HCV) is a single-stranded RNA virus from the genus Hepacivirus and family Flaviviridae. The nonstructural region of HCV includes NS2, the NS3–4A complex, the NS4B, NS5A, and NS5B proteins, and the HCV structural region contains the E1–E2–p7 core proteins. NS5A plays an important role in virus replication and is a hydrophilic phosphoprotein. NS4B is a small hydrophobic protein (27 kDa) that plays an important role in the uptake of other viral proteins. NS5A and NS5B proteins, which are nonstructural HCV proteins, are also degraded by the 26S enzyme. As well as ubiquitylation, NS5A modified with a ubiquitin-like modifier, ISG15 (ISGylation) reduces NS5A protein expression. 24

Zinc mesoporphyrin (ZnMP) increases the polyubiquitylation and proteasome degradation of NS5A, thereby repressing HCV RNA proliferation. The role of ubiquitin-dependent proteasomal degradation in NS5A is unknown. 25

NS5B protein of HCV binds to the uba domain of a ubiquitin-like protein called hPLIC1 and acts as an RNA-dependent RNA polymerase. This results in increased polyubiquitylation and proteasome degradation of NS5B. 26

**Fig. 3:**
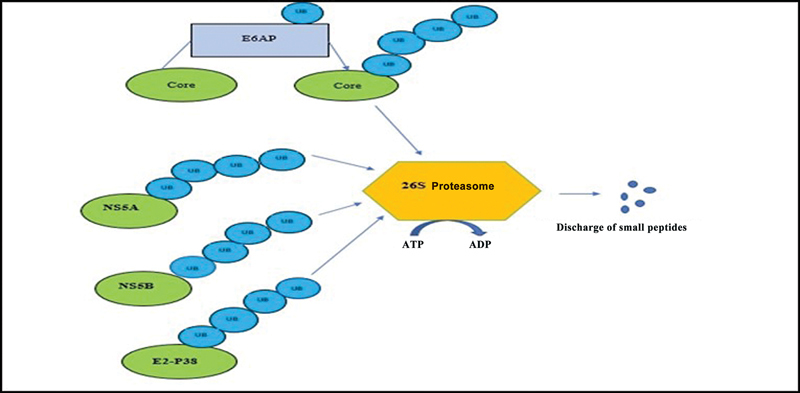
NS5A, NS5B (binding to uba domain), and E2–P38 are degraded by proteasome.

Most HCV proteins, after ubiquitylation, are degraded by the 26S proteasome. HCV envelope proteins, including glycoproteins, E1 and E2. Both these proteins play an important role in cell entry. Various domains of E2 also play important roles in virus entry into the host cell. HCV envelope protein, E2, is phosphorylated by protein kinase R. Unglycosylated forms of protein E2 (E2–p38) are ubiquitylated and degraded via proteasome ( Fig. 3 ). 27

F-protein and NS2 protein are involved in Ub-independent degradation of HCV proteins via the proteasome. F-protein binds to the region between amino acids 40 to 60 in the α3 subunit of the 20S proteasome without polyubiquitination. F-protein is very unstable and is also known as an alternative reading frame protein. 28 29 The NS2 protein is a short-lived protein with protease activity that is quickly destroyed through the activity of casein kinase 2 by the proteasome in a phosphorylation-dependent way. Research shows that HCV NS2 protein degradation is proteasome-dependent and without ubiquitination. 30

In HCV pathogenesis, core protein plays a significant pattern. HCV core protein is regulated by two separate ways; the first pathway is through the PA28γ-related ubiquitin-independent, ATP-independent, and associated with hepatocarcinogenesis. As a result, HCV is positively regulated by PA28γ.

The second pathway is through the E6AP-related ubiquitin-dependent, ATP-dependent, and inhibits virus production. 31

Now, the most important progress has been found in describing the role of Ub-dependent degradation pathways with HCV core protein. In the cytosol, the core protein of hepatitis C is ubiquitylated by E6AP, a type of E3 ligase, and then destroyed. It is responsible for the interaction with E6AP, the conserved area between amino acids 58 and 71 of the HCV core protein. 32

It has recently been shown that only Lys 48-linked polyubiquitin chains do not initiate proteasome-dependent degradation. Also, monoubiquitylation, Lys 11-linked chains, Lys 63-linked chains, and linear chains have been introduced to have different functions, including the cell cycle development and activation of signaling pathways. 33 34

## Hepatitis B

HBV is a small DNA virus containing the 3.2-kb HBV genome that infects human liver cells. The HBV genome consists of four overlapping open reading frames (ORFs), two direct repeats (DR1, DR2) and two enhancer regions (Enh1, Enh2) promoters, and viral amplifiers are placed in ORFs. 35

After attaching and entering the HBV virus into the liver cells, the HBV virus is uncoated, and the viral genome is transferred to the cell nucleus, and the covalently closed circular DNA is formed. Then, at last, the particles of the viral nucleus either obtain an envelope or have been out of the cell or back to the nucleus to start a new replication. 36 The replication of the HBV is modulated by the UPS through the regulation of viral proteins. Np95/ICBP90 (NIRF), like RING finger protein, can bind to HBc and result in increased ubiquitination and decreased HBc protein levels. 37

Inhibitor of apoptosis protein 2 (cIAP2) is capable of HBV protein degradation via the UPS, which has a carboxyl-terminal RING finger domain E3 ligase activity. 38

HBV encodes a 17 kDa HBx protein required for virus replication. The contribution of HBx in virus replication is unknown. HBx has a conserved sequence among mammalian hepatitis viruses. Using a genetic strategy, Leupin et al demonstrated HBx connects to one of the UPS components called damaged DNA binding protein 1 (DDB1) and at the end, HBx interactions with CRL4 (cullin-RING ligase 4) complex, and this result is in interaction with the UPS. 39 The HBx protein of hepatitis B virus binds to the F-box protein Skp2 and prevents the ubiquitination and proteasomal degradation of c-Myc and hence causes the development of hepatocellular carcinoma. 40 Understanding how HBx protein interactions favor the replication of the virus due to the limited models in which the study of HBV replication is still a challenge. Therefore, other studies of viral systems that similarly target the UPS suggest ways of escaping the virus from the UPS. 41

Research has shown that UPS works in two different ways in HBV infection. On one hand, UPS as a host defense mechanism eliminates virus components and destroys the virus life cycle. Several studies have shown that the UPS through selective destruction of viral protein acts as an antiviral mechanism. 37 38 40 On the other hand, the UPS could be used by the HBV to retain the level of viral proteins and to provide viral replication. 42

Several proteins have been identified to control HBV proliferation through interaction with HBx and the mechanism associated with UPS. The Cul4A gene located on human chromosomes q13 and q34 is amplified in liver cancer, suggesting that increased expression of CUL4A may promote carcinogenesis. 43 DNA binding protein 1 (DDB1) and E3 ligase Cullin 4 (CUL4) interact with HBx, forming HBx–DDB1–CUL4 E3 ligase complex, and preserve viral protein against UPS. 41 44

Huang et al, 45 using a *Saccharomyces cerevisiae* two-hybrid system, identified a new subunit of the 20s proteasome (XAPC7) that specifically interacts with HBX. Hbx by activating or deactivating various components of the UPS and ubiquitination of proteins can promote liver cancer.

**Fig. 4:**
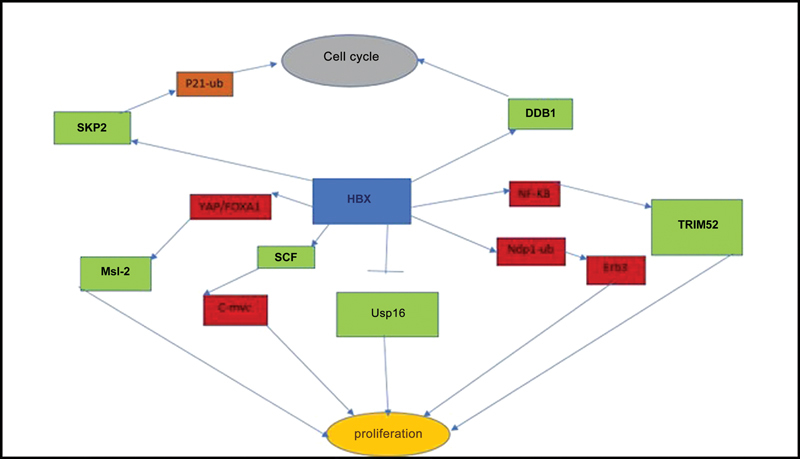
HBx affects some components and then promotes hepatocyte proliferation.

For example, HBX increases the inhibition of usp16 and also activates TRIM52 and MSL 2 (two components of UPS) through NFκB signal and YAP/FoxA signaling, respectively, to promote hepatocyte proliferation. Also, by activating SCF, it inhibits the ubiquitination of c-Myc, resulting in liver cell proliferation. 42 HBx Increases ErbB3 expression by increasing neuregulin receptor degradation protein 1 (Nrdp1) and promotes hepatocellular carcinoma cell proliferation ( Fig. 4 ). In conclusion, the proteasome complex is probably the functional target of HBX in hepatocytes. 42

## Papillomaviridae

Papillomaviridae is a family of small circular double-stranded DNA and nonenveloped viruses with 55 nm in diameter. The morphology of the capsid is icosahedral in which 72 capsomers that are tubular and filamentous formations are observed. The virion is nonenveloped, so it is resistant to some materials including acid, ether, and high temperature (50°C) for 1 hour. 46

Papillomaviridae infects vertebrates, especially humans, that are transferred via close contact and widely distributed in the world. 46 Recent studies have recognized a member of papillomaviridae, *Sparus aurata* papillomavirus 1 (SaPV1), in fish. 47

Most of papillomaviridae containing the circular genome with 6.8 to 8.4 Kbp is known as E1 to E8 (nonstructural proteins) that some members lack the E3 and E8 and L1 to L2 (structural proteins). E1 and E2 proteins bind to the origin of replication and E5, E6, and E7 related to cellular DNA replication. E4 is a late protein that binds to cytoskeleton structures. L1 and L2 are structural proteins and playing an important role in making the capsid. The viral DNA has two forms, the integrated form is in cervical cancers and the episomal form in skin carcinomas. 46 The collaboration of the E6 and E7 genes is vital to keep alive human transformation cells that are infected with HPV. 48

The L1 gene encodes capsid proteins. It is the important particle that is conserved in all known papillomaviruses due to it is suitable candidate using for vaccines and classification. In 2004, de Villiers et al characterized HPV types in phylogenetically, genetically, and biologically areas afterward determined in some genera. According to the Greek alphabet, these genera was determined and confirmed by the International Committee on Taxonomy of Viruses. Number of HPV types were classified in five genera including α (Alpha), β (Beta), γ (Gamma), µ (Mu), and ν (Nu). 49

Human papillomaviruses (HPV) are located in mucous membranes, and they are isolated from microlesions of basal layer cells. 50 The α-HPV is subdivided into, low-risk (HPV-6 and HPV-11) and high-risk (HPV-16, HPV-18, and HPV-33) (International Agency). Low-risk HPV commonly causes benign lesions of squamous epithelial, as warts, and respiratory papillomatosis while high-risk HPV generates malignant diseases, for example, cancers of the anus, vulva, vagina, penis, and oropharynx. 51 52 53 At first, HPV-16 and HPV-18 were isolated from cervical cancer in 1983 and 1984, respectively. 54

Three genes of HPV such as E5, E6, and E7 have a significant role in proliferation of infected cells. For example, the E5 appears at the beginning of infection cells and the important role to form cancer cells is related to E6 and E7. E6 and E7 have more effective functions when they expressed together in cancer cells. 54

Initial experiments showed that E6 targets the cellular p53 and BAK proteins and degrades them. In contrast, E7 binds and degrades the cellular retinoblastoma tumor suppressor protein (pRb) and increases the S-phase genes, cyclin A and E. p53 is a tumor suppressor protein. In mitochondria, BAK and BAX, pro-apoptotic proteins, have been activated by p53, while BCL-2 and BCL-XL, anti-apoptotic proteins can bind and inactive via p53. 48

E6 protein of low-risk and high-risk HPV binds to carboxyl-terminal domain of p53, because the carboxyl-terminal domain of p53 is an early site that identified by E6 protein of low-risk and high-risk HPV, but this binding has no effect on p53. The core domain of p53 is a secondary site that is recognized by E6 protein. Only high-risk E6 protein can interact with the core of p53, E6-associated protein (E6–AP). Hence, high-risk E6 protein can degrades p53, whereas low-risk E6 protein is only able to bind p53 without degradation. 49

A thioester is formed between the cysteine domain of El enzyme and the carboxyl-terminal domain of glycine of free ubiquitin with ATP interfere then the activated ubiquitin is transferred to a cysteine domain of E2 enzymes (ubiquitin-conjugating). 55

**Fig. 5:**
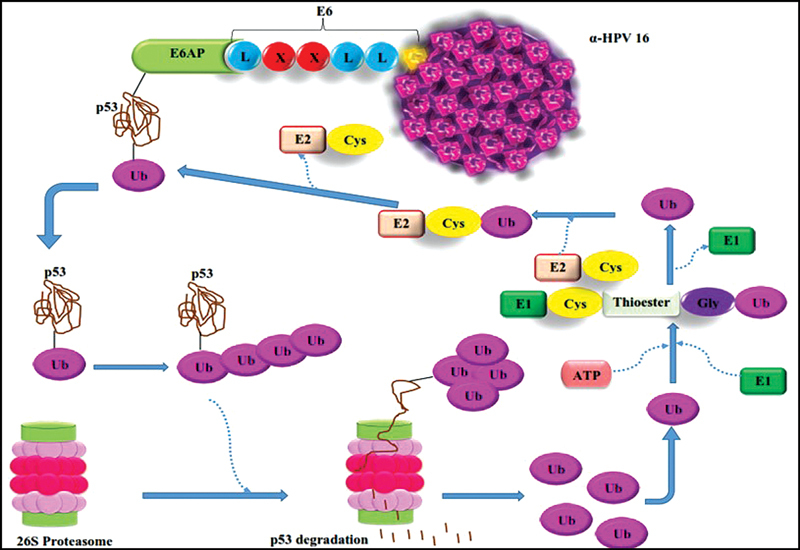
E6 protein of α-HPV 16 binds to p53 by E6AP, then p53 is ubiquitinated and degraded by 26s proteasome, and ubiquitin are released.

After E6 protein of α-HPV-16 interacts to E6-associated protein (E6AP) ubiquitin ligase by LXXLL motif, an LXXLL (where L is leucine and X is any amino acid) binding motif on α-HPV-16 E6 protein, E6 protein prepares to bind p53. Many ubiquitin interact with p53, and afterward polyubiquitinated p53 is degraded by the 26S proteasome ( Fig. 5 ). 55 56 57 58

The retinoblastoma protein (pRb) is a tumor suppressor that cooperates with two proteins, p107 and p130, so it regulates proliferation in cell cycle. Studies have shown that pRb, p107, and p130, as a pocket proteins, are RPs in a gene. The pocket proteins binding to E2F, which encodes transcription factors, inhibit S-phase and effect on differentiation, apoptosis, and other cellular processes. When HPV-16 E7 binds to pRb via the conserved sequence domain, LXCXE (leucine–X–cysteine–X–glutamate) (X is any amino acid) motif on amino terminal HPV-16 E7, the interaction between pRb and E2F has been disordered and affected on cell cycle. Studies demonstrated that, in this pathway, HPV-16 E7 expression induces degradation of pRb, but the mechanism are unknown. 59 60 61

The S4-ATPase is an important subunit in 26S proteasome. The carboxyl-terminal domain of E7 is a zinc finger containing Cys–X–X–Cys motif that binds to S4 and the increases ATPase activity of S4. The interaction between E7 and S4 is independent of the E7–pRb interaction. Studies have shown that pRb might degraded by E7 through interaction with S4 in 26S proteasome. The degradation of pRb is a direct interacting between E7 and S4. The interaction between E6 and p53 has no effect on degradation of pRb by E7. 62 63 64

**Fig. 6:**
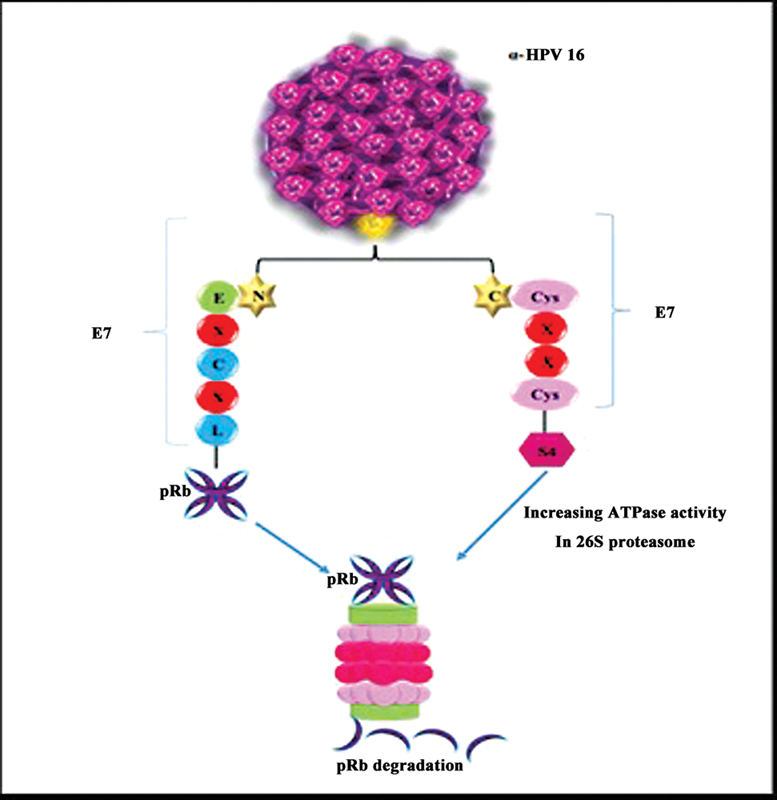
E7 of α-HPV 16 binds to pRb by C- terminal domain. The C-terminal domain binds to S4 for inducing ATPase activity. Also, E7 binds to pRb by N-terminal domain then pRb degrades through proteasome.

E6 and E7 proteins of the low-risk HPV, HPV-6 and HPV-11, might act in similar way that showed in the high-risk proteins ( Fig. 6 ). 65

## Polyomaviridae

Polyomaviridae has a circular genome, double-stranded DNA (dsDNA), nonenveloped with 40–45 nm in diameter. The morphology of the capsid is icosahedral in which is 72 capsomers are observed. Similar to papillomaviridae, the virion is resistant to ether and high temperature (50°C) for 1 hour. Likewise, polyomaviridae is similar to papillomaviridae in morphology of the capsid and components of nucleic acid. 46

Polyomaviridae infects vertebrates. Transmission of polyomaviridae may relate to organ transplantation in human. 46 Merkel cell polyomavirus (MCPyV) was discovered as inducing factor of Merkel cell carcinoma (MCC) by Chang in 2008. MCC tumors progresses in the skin and follicles of hair. Infection caused by active MCPyV seems asymptomatic in individuals with healthy immunity, otherwise MCC. The route of transmission is not precisely clear, but it could transfer via close cutaneous contacts. 66

The first human malignancy related to polyomavirus is MCPyV that lately discovered in human MCC through relating integrated DNA between them. The only polyomavirus that can integrate into the human genomic DNA is MCPyV. In approximately 80% of MCC is seen the MCPyV genome 67 and the genomic sequence of the large T antigen (LT-antigen) of MCPyV is mutated in MCC. 46

The genome size of polyomaviridae is 4.7 to 5.4 kbp. Tumor T antigens (T-Ag) are nonstructural proteins, because they regulate cell cycle progression and provoke tumorigenesis. Three structural proteins are Vp1, Vp2, and Vp3. Vp1 is a major structural protein in virion and Vp2 and Vp3 interfere in encapsidation of the replicated polyomavirus genome. In addition, Vp2 plays a role in entry to host cells. In polyomaviridae, nonstructural proteins and structural proteins are named as agnoprotein. 46

Indeed, the MCC tumors express MCPyV small T antigen (ST-antigen). It seems ST-antigen has a tumorigenesis function. The ST-antigen has an LSD domain (L stabilizing domain) that elevates the level of LT antigen and viral replication. 68 The LSD of ST-antigen interacts to the cellular ubiquitin ligase SCF ^Fbw7^ and inhibits proteasomal degradation of LT-Ag, so interacting between ST-antigen and SCF (E3 ubiquitin ligase)-induced tumorgenesis. 69 70

SCF (Skp, Cullin, F-box) is one of the E3 ubiquitin ligases that regulates the proteins include in cell cycle progression. 68 SCF ubiquitin ligase contains an Fbw7 that detects and uses substrate for ubiquitinating proteins. SCF ^Fbw7^ interacts and ubiquitinates the proteins, thereby the proteins degraded by proteasome. 71

The LT-antigen of MCPyV has important roles in transformation of the host cell and viral replication. LT-antigen is conserved in all of polyomavirus. 66 LT-antigen interacts to pRb with LXCXE motif on its amino-terminal and dysregulates E2F, then induces cells into S-phase. Indeed, in the carboxyl-terminal of LT-antigen are OBD and zinc-finger motif that have a role in LT replication. Studies showed that MCV LT-antigen can interfere p53 with indirect interaction. 72

**Fig. 7:**
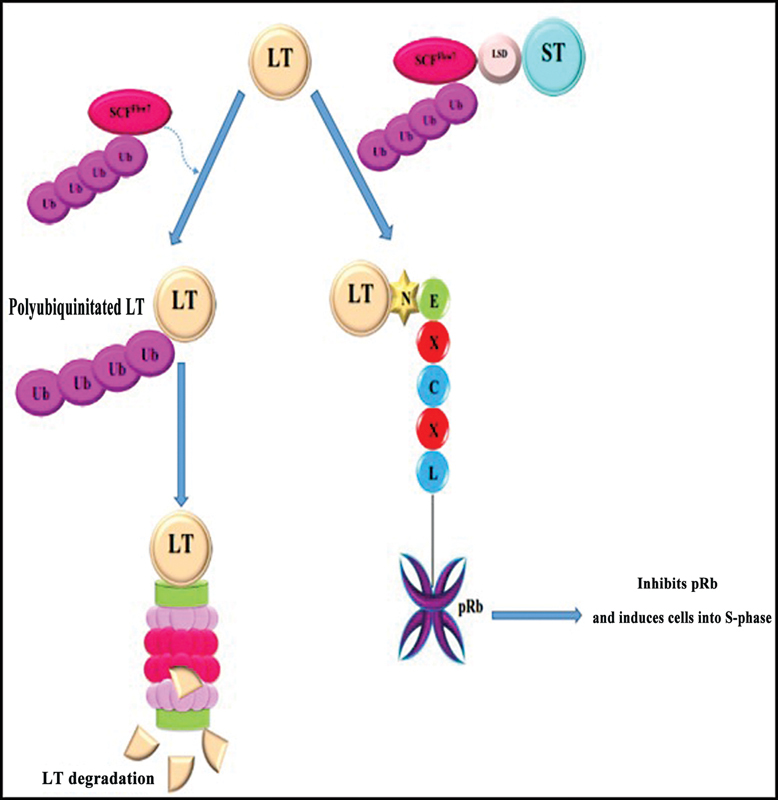
LT-antigen binds to SCF ^Fbw7^ and degrades through proteasome. After binding ST-antigen to SCF ^Fbw7^ , LT-antigen can bind to pRb, then degrade it by proteasome and lead the cell to S-phase.

LT-antigen induced antitumorigenic effects of p53, whereas ST-antigen has inhibiting function on p53, so in host cells, LT-and ST-antigens co-expressed and alleviated activity of p53 ( Fig. 7 ). 70

## Herpesviridae

Herpesviridae is a family of small linear segment double-stranded DNA and an enveloped viruses with 160 to 300 nm in diameter. The morphology of the virion is spherical, and the genome size is 125 to 241 kbp. The persistence of different Herpesviridae divers, but they are generally sensitive to low pH. In addition, the enveloped virion sensitive to detergents. The virion uses membrane proteins in its envelope for entering into host cells. 46

Herpesviridae genomes involved ORFs that encoded proteins. In addition, the genomes included many microRNA genes. These genes encoded capsid, membrane, and tegument proteins and have the necessary components in replicating DNA and modifying enzymes. A lot of host species infected by Herpesviridae. It is possible the whole of vertebrates carry Herpesviridae. 46

According to biological characterization Herpesviridae includes three subfamilies: *Alphaherpesvirinae* , *Betaherpesvirinae* , and *Gammaherpesvirinae* . KSHV is a member of gammaherpesvirus, γ2-lymphotropic-oncogenic virus belongs to *Rhadinovirus* genus. 46 In 1872, KS lesions initially called idiopathic multiple pigmented sarcoma of the skin, was first described by Moritz Kaposi, a Hungarian dermatologist. After decades, Antman, another dermatologist, suggested idiopathic multiple pigmented sarcoma of the skin as a KS. 73 In 1994, DNA of a KS was isolated by Mesri et al in an AIDS patient. KS shows one of the most mortality in AIDS patients. 74

KSHV known as human herpesvirus 8 (HHV-8). It is one of the lately discovered human tumor viruses known human γ2-herpesvirus and it is related to EBV. 75 KSHV is related to KS and B-cell diseases, primary effusion lymphoma, and multicentric Castleman's disease. 76

KS is atypical neoplasm differed from other usual tumors and recognized by dark purple lesions. KS is recognized as a slow tumor with malignancy that contributes to individuals with immunodeficiency; the virus is in latent infection. 77 78

The genes of KSHV divided in two groups, including latent infection and lytic infection genes that regulate by KSHV. The number of viral proteins expressed in latent infection are LANA (latency-associated nuclear antigen), viral cyclin, and viral FLIP encoded by ORF73, 72, and 71, respectively. 79

During latent infection, one of the proteins in all forms of KSHV is LANA that encoded by ORF73. 80 LANA plays an important role in cellular functions. Significantly, LANA interacts with some tumor suppressor protein (p53) and G1–S checkpoint proteins (pRb), by the carboxyl-terminal domain, and it inhibits pRb- and p53-mediated apoptosis. LANA causes increased cell cycle progression, transcripting and replicating viral episomal DNA for maintenance and immortalize cell. Previous studies showed that the amino-terminal of LANA has no ability interaction with p53 and does not effect on cell cycle progression. In addition, LANA enables bind to pRb by carboxyl-terminal domain. 81 Thus, LANA has several functions. 74 79 82 83

Notably, cytoskeletal proteins were proteins that are related to the carboxyl-terminal, so it suggests LANA is related to structural proteins. Studies showed that LANA can dysregulate genes that LANA strongly interacts with different factors that role in tumorigenesis or tumor suppression. 79

The domain of LANA binds to p53 is composed of the 400 amino acid sites in the carboxyl terminal. After inhibiting of pRb and p53, the cells expression LANA escape from apoptotic pathway and immortalized cells. In deregulation of cell cycle, LANA is an important KSHV protein, similar to HPV E6 and E7 proteins.

**Fig. 8:**
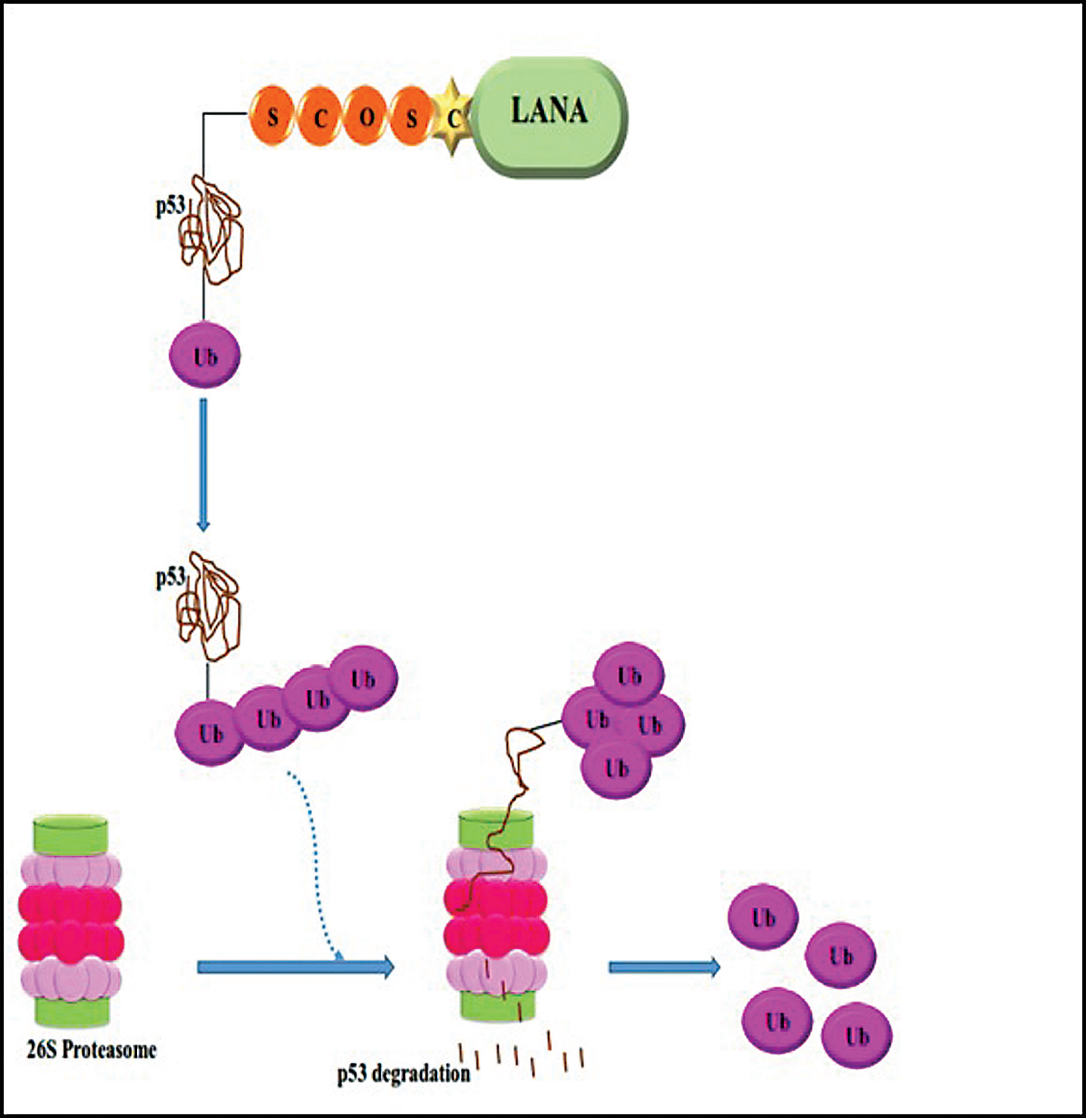
LANA protein of KSHV can bind to p53. p53 is ubiquitinated and degraded through 26S proteasome.

Suzuki et al demonstrated that LANA is the E3 ubiquitin ligase complex containing Elongin BC and Cul5/Rbx that are responsible for diagnosis of p53, then induced p53 ubiquination; afterward polyubiquitinated p53 is degraded by the 26S proteasome. 84 Cai et al demonstrated that LANA can bind to p53 by SOCS (suppressors of cytokine signaling) motif, so polyubiquitinated p53 is degraded by the 26S proteasome. 85 Lack of p53 increased cell cycle progression. Therefore, LANA is important for viral maintenance by inducing cell cycle progression and dysregulating p53. Studies showed that during latent infection, the important mechanism for oncogenesis of KSHV is control of protein degradation ( Fig. 8 ). 84

## Retroviridae

Retroviridae is a member of single-stranded RNA (ssRNA) and enveloped viruses with 80 to 100 nm in diameter. The morphology of the capsid is spherical. The virion is enveloped, so it is sensitive to antimicrobial agents (formaldehyde, detergents, etc.) and high temperature but resistant to UV light. 46

The linear genome characteristic of Retroviridae is ssRNA, positive sense with 7 to 13 kb in size, whereas the genome size of *Deltaretrovirus* is approximately 8.3 kb. Tax and Rex are nonstructural genes, which have an important role in the synthesis. The gag, pro, pol, and env are structural genes. Two envelope proteins that are encoded by env gene are surface (SU) and transmembrane (TM). 46

Retroviridae is divided into two subfamilies: Orthoretrovirinae and Spumaretrovirinae. Orthoretrovirinae was classified into six genera including *Alpharetrovirus* , *Betaretrovirus* , *Gammaretrovirus* , *Deltaretrovirus* , *Epsilonretrovirus* , and *Lentivirus* . 46

The human T cell leukemia virus type 1 (HTLV-1) is a species of the genus *Deltaretrovirus* that was discovered by Gallo and Gonçalves et al from T cell line of a patient with cutaneous T cell lymphoma in the United States in 1979 and submitted in 1980. 86 87 HTLV-2 was discovered in Central and West Africa; North, Central, and South America; United States; and Europe and has a homology with HTLV-1. 88 After the discovery of HTLV-1 and HTLV-2, two more related viruses, HTLV-3 and HTLV-4, were described in Central Africa. 89 Only HTLV-1 has been related to human diseases. 87 This virus carries double-stranded RNA and the virion of HTLV-1 has an envelope and capsid. The capsid includes reverse transcriptase, integrase, and functional protease. It is 100 nm in diameter. 90

Retroviridae infects vertebrates. Only HTLV-1 has been related to human disease. The HTLV-1 is a species of the genus *Deltaretrovirus* . 46 Although HTLV-1 mostly causes asymptomatic infections, the infectivity mechanism is unknown. Studies showed that HTLV-1 infections are related to T-cells, especially CD4 + T lymphocytes. ATL is a scarce and fast-growing malignancy of T cell lymphoma that can be seen in the lymphoma nodes and in the blood known as a leukemia. Less than 5% of HTLV-1 infections developed to ATL. In addition, neurological disease that is related to HTLV-1 is tropical spastic paraparesis or HTLV-associated myopathy. Furthermore, HTLV-1 can be found in B-cells, CD8+ T-cells, dendritic cells, and monocytes as reservoirs of infected cells. 91 92 93 94 95

Three different transmission routes used by HTLV-1 are breastfeeding as a vertical transmission, sexual contact as a horizontal transmission, sharing needles or syringes for injecting drugs, transferring blood or blood products, and organ transplantation. These routes refer to a mechanism that depends on cell-to-cell transfer. 96 97 98 99 100 In cell-to-cell transmission of HTLV-1, Tax has an important role. Tax is between cell harbor HTLV-1, as a donor cell, and target cell. In addition, Tax is related to Golgi. 96

The HTLV-1 oncoprotein, Tax, is produced in the cytosol, then penetrated to the nucleus, but the mechanism of transfer is unknown. Tax strongly dysregulates genes expression, which have a role in cell proliferation, cell cycle regulation, transcription, and apoptosis by physical interactions, resulting in cellular transformation and the advancement to ATL. Although Tax can be affected on the cellular functions, studies showed that Tax has no effect on DNA damage development. 101 102 Studies showed that lysines involving K263, K280, and K284 are in the carboxyl-terminal domain of Tax, which keeps its stability. Tax binds to 26S proteasome through lysine' domains without degradation; so this interaction suggests that Tax does not have proteolytic functions. 103

**Fig. 9:**
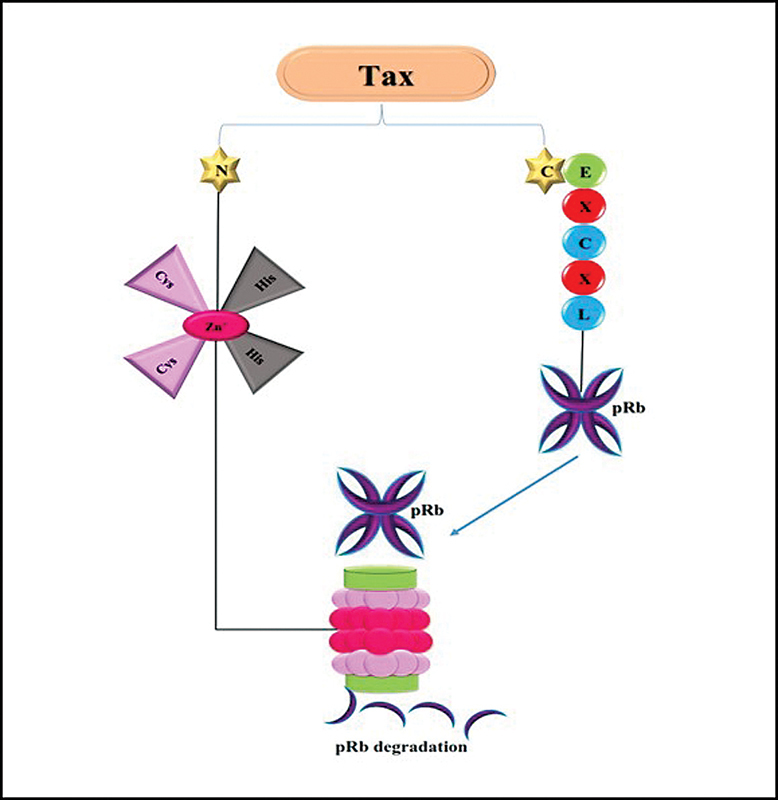
Tax protein of HTLV binds to pRb by C-terminal domain and binds to proteasome via zinc-ﬁnger-binding motifs. Afterward, pRb degrades.

The zinc-ﬁnger-binding motifs on amino-terminal domain of Tax interact to 26S proteasome. In addition, Tax binds to pRb (the B pocket and the carboxy-terminus) through a conserved LXCXE motif. It showed that Tax can be a bridge between pRb and proteasome, so the pRb degrades. The degradation of pRb results in inducing cellular processes ( Fig. 9 ). 104

## Conclusion

The critical strategies used by tumor viruses to create an optimal environment in which they can successfully replicate is the interplay between oncoproteins from viruses and the UPS. For example, E6 and E7 ( *Papillomaviridae* ), LT and ST-antigen ( *Polyomaviridae* ), LANA ( *Herpesviridae* ), Tax ( *Retroviridae* ), EBNA-3C and EBNA-1 ( *EBV* ), NS5A, NS5B, and E2P38 ( *HCV* ) and HBX ( *HBV* ) are viral oncoproteins that interplay to UPS. As seen in the Figs. 1 , 2 , 3 , 4 , 5 , 6 , 7 , 8 , 9 , interacting between viral oncoproteins and UPS can modulate cellular functions and eventually lead to cancer of the cell. It seems that viral oncoproteins, which are associated with various human cancers at different anatomical locations, have shown interacting with many of the UPS components. This interaction affected the process of tumorigenesis. Therefore, developing novel strategies for the interaction between viral oncoproteins and UPS components, it may happen to be designing new therapies against tumorigenesis viruses.
